# MiR‐193b deregulation is associated with Parkinson's disease

**DOI:** 10.1111/jcmm.16612

**Published:** 2021-05-20

**Authors:** Masoud Baghi, Elaheh Yadegari, Mahsa Rostamian Delavar, Maryam Peymani, Mazdak Ganjalikhani‐Hakemi, Mehri Salari, Mohammad Hossein Nasr‐Esfahani, Timothy L. Megraw, Kamran Ghaedi

**Affiliations:** ^1^ Department of Cell and Molecular Biology and Microbiology Faculty of Biological Science and Technology University of Isfahan Isfahan Iran; ^2^ Department of Animal Biotechnology Cell Science Research Center Royan Institute for Biotechnology ACECR Isfahan Iran; ^3^ Department of Biology Faculty of Basic Sciences Shahrekord Branch Islamic Azad University Shahrekord Iran; ^4^ Immunology Department School of Medicine Isfahan University of Medical Sciences Isfahan Iran; ^5^ Functional Neurosurgery Research Center Shohada Tajrish Neurosurgical Center of Excellence Shahid Beheshti University of Medical Sciences Tehran Iran; ^6^ Department of Biomedical Sciences Florida State University College of Medicine Tallahassee FL USA

**Keywords:** miR‐193b, MPP+, Parkinson's disease, peripheral blood mononuclear cells, PGC‐1α/FNDC5/BDNF pathway, SH‐SY5Y cells

## Abstract

PGC‐1α/FNDC5/BDNF has found to be a critical pathway in neurodegeneration. MicroRNAs (miR(NA)s) are non‐coding regulatory RNAs whose dysregulation has been observed in multiple neurological disorders, and miRNA‐mediated gene deregulation plays a decisive role in PD. Here, candidate miRNA was chosen based on the literature survey and in silico studies. Chronic and acute models of PD were created using MPP+‐treated SH‐SY5Y cells. Twenty PD patients and 20 healthy volunteers were recruited. RT‐qPCR was performed to assess the expression of miRNA and genes. Severe mitochondrial dysfunction induced by acute MPP+ treatment instigated compensatory mechanisms through enhancing expression of PGC‐1α/FNDC5/BDNF pathway genes, while chronic MPP+ toxicity led to down‐regulated levels of the genes in SH‐SY5Y cells. PD peripheral blood mononuclear cells (PBMCs) also showed decreased expression of target genes. There were significant changes in the level of miR‐193b in both models, as well as PD PBMCs. Moreover, miR‐193b overexpression significantly affected PGC‐1α, FNDC5 and TFAM levels. Interestingly, down‐regulations of PGC‐1α, FNDC5, BDNF and TFAM were inversely correlated with miR‐193b up‐regulation in PD PBMCs. This study showed the deregulation of PGC‐1α/FNDC5/BDNF pathway in PD models and PBMCs, verifying its importance in neurodegeneration. Our findings also revealed that miR‐193b functions in PD development, possibly through regulating PGC‐1α/FNDC5/BDNF pathway, suggesting miR‐193b as a potential biomarker for PD diagnosis.

## 1 INTRODUCTION

Parkinson's disease (PD) is a progressive neurodegenerative disorder (NDD) caused by the progressive and selective degeneration of dopaminergic neurons within the substantia nigra pars compacta (SNpc) as well as accumulation of Lewy bodies containing α‐synuclein in neocortex and brainstem.^1^, ^2^, ^3^ While the precise mechanisms underlying pathogenesis of PD are not yet fully understood, several molecular mechanisms comprising oxidative stress, abnormal protein aggregation, mitochondrial dysfunction and neuroinflammation have been found to be strongly implicated in its pathogenesis, leading to programmed cell death of neurons.^3^, ^4^, ^5^, ^6^, ^7^ 1‐methyl‐4‐phenylpyridinium (MPP+) is a complex I activity inhibitor extensively utilized to induce PD chemically due to its potential to create mitochondrial impairment followed by oxidative stress and apoptosis, all of which have close associations with PD pathogenesis.^2^ ^,^ ^4^

Besides environmental factors playing an indispensable role in PD pathology, some susceptibility or causative genes and pathways have been discerned in PD,^1^, ^7^ Xi.^8^ PGC‐1α/FNDC5/BDNF pathway is one of them whose importance in neuroprotection as an effective target in neurological diseases and ageing has been highlighted.^4^ Peroxisome proliferator–activated receptor gamma coactivator 1‐alpha (PGC1α) has been identified as a master regulator of mitochondrial biogenesis and respiration that preserves neurons against degeneration.^4^, ^7^ It has been reported that PGC‐1α overexpression positively regulates the expression of genes required for mitochondrial biogenesis such as TFAM and antioxidant capacity via rising expression of ROS‐detoxifying enzymes, whereas PGC‐1α silencing suppresses the expression of antioxidant enzymes as well as mitobiogenesis regulators. Thereby, PGC‐1α pathway can be considered as a critical point of therapeutic intervention for PD and other neurological disorders.^4^, ^9^ Mitochondrial transcription factor A (TFAM) is a key modulator of the replication and transcription of mitochondrial DNA whose expression can be stimulated by PGC‐1α.^10^ Studies have shown possible connections between TFAM as a multifunctional regulator of mtDNA metabolism and PD pathogenesis.^11^ There are many lines of evidence indicating that BDNF/TrkB is closely associated with pathophysiology of many NDDs^12^, ^13^, ^14^ and enhanced expression of it has been demonstrated to defend striatal neurons against neurotoxicity in several PD models.^4^ Brain‐derived neurotrophic factor (BDNF) has been recognized as a versatile and multifunctional neurotrophin playing a vital role in a wide variety of neuronal functions ranging from neurogenesis and survival to synaptic plasticity by activating a particular receptor named tropomyosin receptor kinase B (TrkB).^12^, ^15^, ^16^ TrkB is a membrane tyrosine kinase receptor mediating BDNF function which abounds in some brain regions where BDNF is highly expressed.^4^, ^14^ Numerous studies previously have shown that BDNF induces various intracellular signalling events via TrkB receptors, leading to an elevation in PGC‐1α and TFAM expressions.^4^, ^15^ Fibronectin type III domain‐containing protein 5 (FNDC5) is a PGC‐1α‐dependent myokine that induces the expression of neuroprotective agents including BDNF in the hippocampus, culminating in an elevated expression of PGC‐1α in neurons.^4^, ^16^, ^17^ Therefore, as our previous studies suggested, FNDC5 plays a beneficial role in neuronal survival, development, differentiation and resistance to neuronal stress and mitochondrial biogenesis.^4^ ^,^ ^17^ ^,^ ^18^

MicroRNAs (miR(NA)s) are highly conserved endogenous small (~22 nucleotide) non‐coding RNAs that control gene expression at the post‐transcriptional level by binding to the 3' UTRs of mRNAs. ^2^ ^,^ ^19^, ^20^ Multiple lines of evidence confirmed an essential role of miRNAs in neuronal survival and normal function.^21^, ^22^ miRNAs participate extensively in a number of neurodegenerative processes like PD and their dysregulation has been observed in several PD models and post‐mortem human brain tissues, resulting in their being introduced as attractive therapeutic targets or biomarkers for PD.^2^ ^,^ ^20^ ^,^ ^21^ ^,^ ^23^ ^,^ ^24^

Therefore, our study is primarily aimed to assess miR‐193b levels in PD patients’ peripheral blood mononuclear cells (PBMCs) and SH‐SY5Y cells injured acutely and chronically with MPP+, as in vitro models of the disease, in the hope of finding a potential candidate for PD biomarker. Furthermore, since altered expression of miRNAs can profoundly affect PD‐related genes’ expression and disturbed miRNA‐mediated post‐transcriptional regulation is deemed to be an important mechanism in neurodegeneration,^20^ ^,^ ^21^ we checked the expression of PGC‐1α/FNDC5/BDNF pathway components as miR‐193b's potential targets in two PD models and PBMCs.

## 2 MATERIALS AND METHODS

### 2.1 Sample collection and separation of PBMCs

Twenty patients recognized with PD attending Al‐Zahra Hospital (Isfahan, Iran) were selected. The written informed consent was obtained from all studied patients, and the study was performed under guidelines approved by the review board of the Royan Institute and University of Isfahan. The protocol of study to use human samples was confirmed by both the Bioethics Committee of University of Isfahan and the ROYAN institute review board under the bioethical code number: IR.ACECR.ROYAN.REC.1397.216. The severity and stage of the PD patients were evaluated using the Hoehn and Yahr staging scale^25^ and verified by an expert neurologist. Twenty age‐matched healthy volunteers without family history of PD or symptoms of NDDs were also recruited. In order to rule out any confounding factors before clinical assessment, the patients did not take anti‐parkinsonian medications for at least 12 hours. Additional demographic and sample characteristics of PD and control patients are described in Table 1.

**TABLE 1:** Demographic and clinical characteristics of the PD patients and healthy controls

	PD	Control	*P*‐value
No. of patients	20	20	‐
Age (years)	61.7 ± 12.55	58.45 ± 9.39	.372^a^
Gender (M/F)	12/8	14/6	.507^b^
Hoehn and Yahr stage (range)	2.3 ± 0.95 (1‐4)	‐	‐
Disease duration (year)	7.39 ± 5.71	‐	‐

Note

The data are presented as mean ± SD.

^a^ *P*‐value was calculated using Student's *t* test.

^b^ *P*‐value was calculated using the chi‐square test.

After getting the written informed consent, blood samples were drawn from the human donors in tubes containing EDTA (5 mL) and PBMCs were separated by the Lymphodex (Inno‐Train) density gradient centrifugation. Fresh blood samples were diluted in PBS (1:1v/v), gently layered on the Ficoll solution at room temperature and centrifuged at 800 × *g* for 30 minutes. The PBMC‐containing layer was collected and washed twice in PBS at 4°C (250 × *g* for 10 minutes) and the pellet (PBMCs) was stored at −70°C until further usage.

### 2.2 Candidate miRNA selection and signalling pathway enrichment analysis

To perform signalling pathway enrichment analysis, KEGG 2019 and Reactome 2016 were recruited on the ‘Enrichr’ platform (http://amp.pharm.mssm.edu/Enrichr/). Interrelation between selected genes and signalling pathways was also assessed using plug‐in ClueGO v2.3.3^26^ in Cytoscape 3.7.2.^27^ TargetScan 7.1, miRmap and RNAhybrid^28^, ^29^, ^30^ were all served to predict a common miRNA targeting the PGC‐1α/FNDC5/BDNF pathway components. Literature mining was also carried out to find deregulated miRNAs in various NDDs. A human PD microarray data set (GSE16658) was analysed in this study (available on NCBI Gene Expression Omnibus (GEO)). The differential expression of array data has been done via limma^31^ and GEOquery^32^ packages in R. The clustering of differential expression of miRs was done and visualized by using the R package pheatmap.^33^ Hierarchical clustering has been used in this study as the best method for analysing high‐throughput expression data. Additionally, DianaTools MirPath v.3^34^ was employed to visualize the signalling pathways in which miR‐193b is implicated. miRTarBase^35^ was used to find the secondary structure of pre‐miR‐193b. Using TargetScan 7.1 and miRmap, the putative miR‐193b binding sites within five genes implicated in PGC‐1α/FNDC5/BDNF pathway were retrieved.

### 2.3 Cell culture

SH‐SY5Y human neuroblastoma cells (Royan Institute, Tehran, Iran) were maintained in the Dulbecco's modified Eagle's medium (DMEM) (Biowest) supplemented with 10% (v/v) foetal bovine serum (FBS), 100 units/mL penicillin/streptomycin, 2 mM L‐glutamine and 1% (v/v) non‐essential amino acids (all from Gibco), at 37°C and in a humidified atmosphere containing 5% CO2 and 95% air. The cells were harvested and dispersed in 80% to 90% cell confluency and were maintained in the log phase during the experiments.

### 2.4 Cell transfection

MiR‐193b mimic and miRNA negative control (scramble) were purchased from Dharmacon Inc SH‐SY5Y cells were plated in 6‐well plates and incubated until 80% confluence prior to transfection. Transfection of oligonucleotides into SH‐SY5Y cells was performed with Lipofectamine 2000 reagent (Invitrogen) using 25 nM of miR‐193b mimic and scramble, followed by treatment with 2000 µM MPP+ for 24 hours. Transfection efficiency was evaluated by qRT‐PCR.

### 2.5 MPP+ treatment and cell viability assessment

In brief, SH‐SY5Y cells were seeded at a density of 2 × 10^4^ cells/cm^2^ for acute and chronic MPP+ treatment. In acute MPP+ model, 24 hours after plating, the medium was replaced with the fresh medium with low serum containing 0‐3000 µM MPP+ (Sigma‐Aldrich) for 24 hours. To create chronic MPP+ model, the cells were exposed to a low‐serum fresh medium containing the same concentrations of MPP+ 3 times per week for up to 2 weeks. Cell viability was assessed using MTS/PMS (phenazine methosulphate). Following neurotoxin treatment, 20 µL of MTS/PMS solution (Promega) was added to each well of 96‐well plates and incubated for 4 hours at 37°C in a 5% CO_2_ atmosphere. The wells were then evaluated for colorimetric absorption with the ELISA microplate reader (Awareness Technology Inc) at a wavelength of 490 nm.

### 2.6 Intracellular ROS measurement

Levels of intracellular ROS were quantified by 2',7'‐dichlorodihydrofluorescein diacetate (DCFH‐DA) assay. DCFH‐DA as a fluorescent dye is de‐esterified intracellularly and turned to a redox sensitive dye, DCFH, that is then oxidized by ROS to 2′,7′‐dichlorofluorescein (DCF), a highly fluorescent probe 17. Following treatment with MPP+, SH‐SY5Y cells were washed with phosphate buffered saline (PBS) (Gibco) and incubated with 0.5 μM DCFH‐DA (Sigma‐Aldrich) in the dark for 20 minutes at 37˚C. Subsequently, the cells were washed and ROS levels were measured using the FACSCalibur flow cytometer (Becton‐Dickinson).

### 2.7 Detection of cell apoptosis by Annexin V staining

The Phosphatidyl Serine Detection^™^ kit (IQ products) was used according to the manufacturer's instructions to detect the apoptotic cells by flow cytometry. SH‐SY5Y cells treated acutely and chronically with toxin were washed by calcium buffer and incubated with 10 µL Annexin V‐FITC for 20 minutes at 4˚C, in the dark, and analysis was performed by the FACSCalibur flow cytometer (Becton‐Dickinson).

### 2.8 RNA extraction and RT‐qPCR analysis

Total RNA was isolated from SH‐SY5Y cells or PBMCs by TRIzol reagent (Invitrogen) and treated with DNaseI (Thermo Scientific), according to the manufacturer's instructions. To synthesize the complementary DNA (cDNA) of miR‐193b, miRCURY LNA^™^ Universal RT cDNA Synthesis Kit (Exiqon/Qiagen) was used. Real‐time quantitative PCR (RT‐qPCR) was done by adding cDNA products to a master mix comprising 2 U of SYBR green Gene Expression Master Mix (TaKaRa) and 10 pmol/μL of the miR‐193b specific primer (Exiqon/Qiagen). cDNA synthesis for PGC‐1α, TFAM, FNDC5, TrkB and BDNF was performed by the cDNA synthesis Kit (TaKaRa) using random hexamer primers. RT‐qPCR was carried out in the ABI PRISM 7500 instrument (Applied Biosystems). Gene‐specific primer pairs were designed by the Beacon designer (Version 7.2) and purchased from Pishgam Company (Pishgam co., Tehran, Iran). The primer pairs are listed in supplementary Table 1. The expressions of miR‐193b and all target genes were normalized to expression levels of U6 small nucleolar RNA and glyceraldehyde‐3‐phosphate dehydrogenase (GAPDH), respectively. All measurements were repeated in three independent experiments. Melt curves after amplification showed that primer pairs generated single products. The data were analysed and reported according to the comparative ΔΔCt method.

### 2.9 Statistical analysis

Statistical analyses were performed by Mann‐Whitney test, Student's *t* test, the chi‐square test using SPSS 22.0 and GraphPad Prism v8.01 (GraphPad Software, Inc). Receiver operating characteristic (ROC) curve analysis and Spearman correlation test were used to assess the diagnostic importance of the miR for discriminating PD patients from controls and its correlation with target genes. All data are presented as the mean ± standard deviation (SD) and collected from three independent experiments. *P*‐value ≤.05 was considered to be statistically significant.

## 3 RESULTS

### 3.1 Characteristics of study patients

Demographic characteristics and clinical data of all participants are presented in Table 1. Statistical analysis showed no significant disparities between two groups in terms of age and sex.

### 3.2 Candidate miRNA selection and PGC‐1α/FNDC5/BDNF pathway enrichment analysis

**FIGURE 1:**
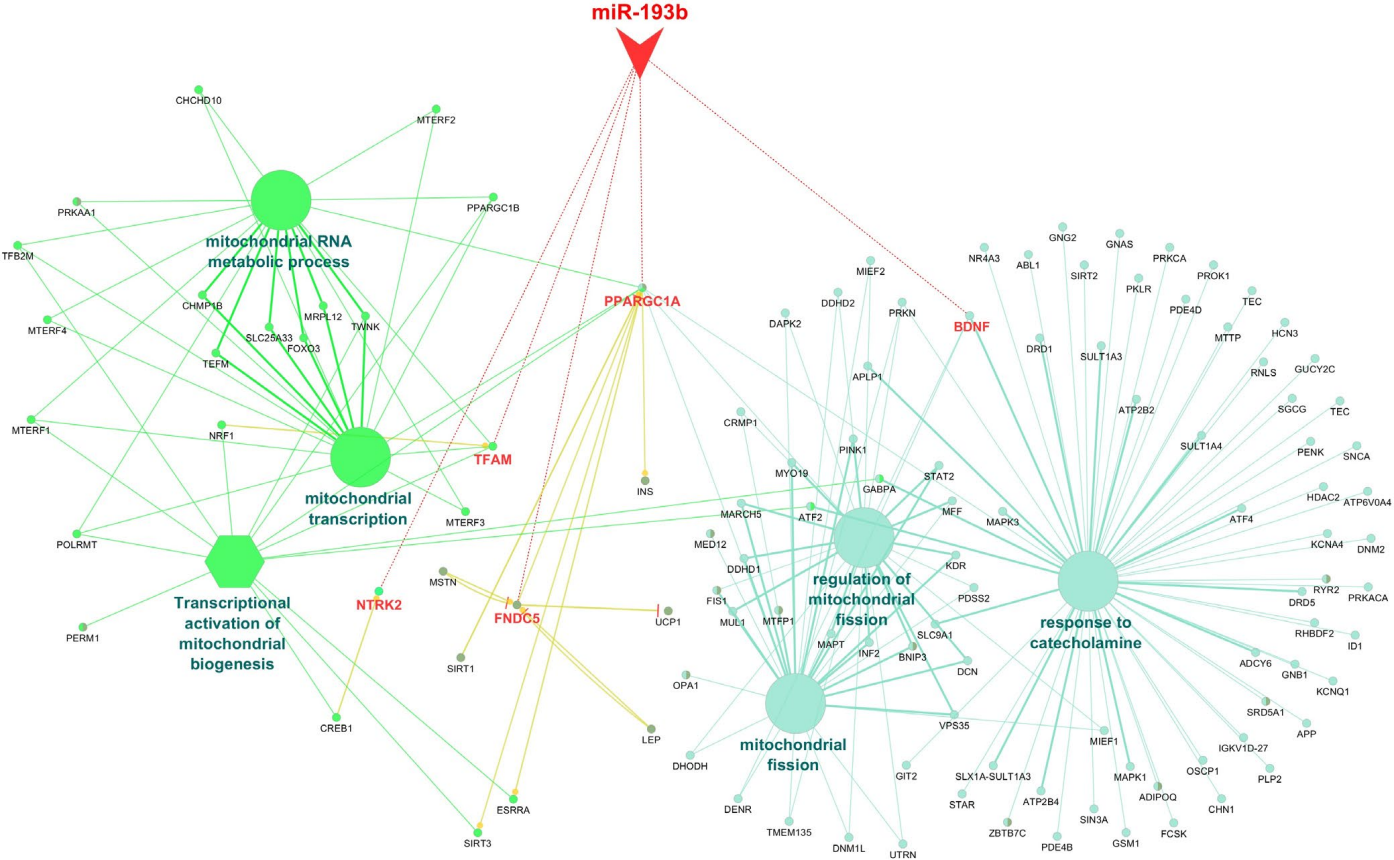
Enriched gene ontology and Parkinson's disease pathways generated by five selected genes. Enriched gene ontology and pathways (in GO and KEGG) have been visualized in the ClueGO app. Each node demonstrated the biological process in their pathways (the significant pathways were shown). The colour of each node represents the functional group that they belong to it. Edges demonstrate the gene‐term, term‐term and miR‐gene interactions. Red lines show interactions of miR‐193b and possible target mRNAs

**FIGURE 2:**
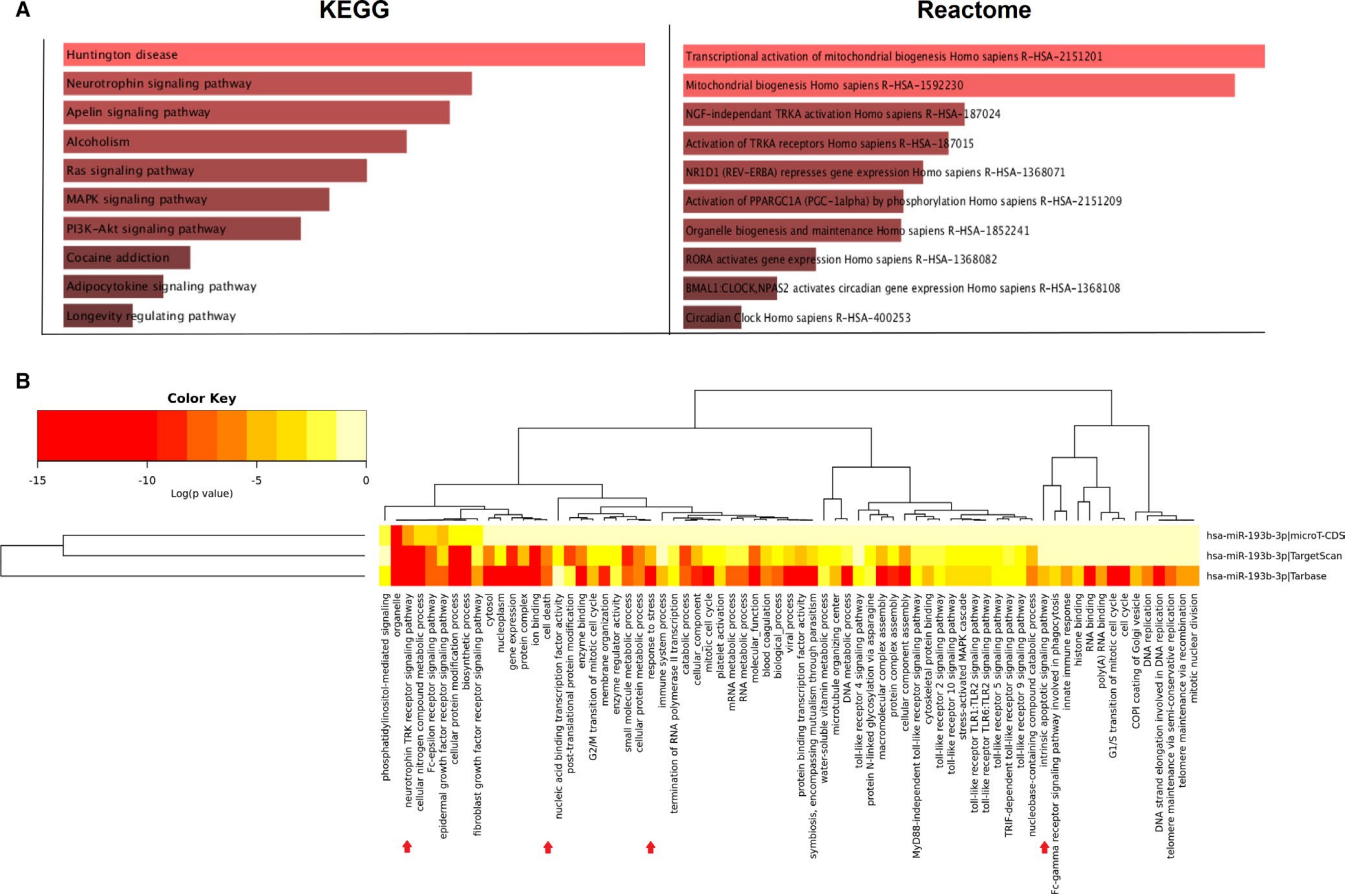
Heatmap view of miR‐193b‐related signalling pathways and pathway analysis of 5 selected genes. A, Pathway analysis of 5 selected genes using KEGG and Reactome libraries. The top 10 enriched pathway terms are displayed in a bar graph ranked by a combined score calculated by *P*‐value. The length of the bar and the brightness of its colour represent the significance of the specific pathway. B, The heatmap demonstrates miR‐193b‐associated pathways according to DIANA miRPath v.3. Here, *P*‐value marks the inspected signalling pathways which are significantly enriched with miRNA targets. Additionally, colour gradient represents pathway value with red, as the highest importance, and pale yellow, as the lowest

Pathways analysis of 5 selected genes (PGC‐1α, BDNF, TFAM, FNDC5 and TrkB) indicated that each of the genes inside the pathway can be associated with some other crucial genes and enriched in some of the following pathways: response to catecholamine, regulation of mitochondrial fission, mitochondrial transcription, mitochondrial RNA metabolic process, transcriptional activation of mitochondrial biogenesis and mitochondrial fission pathways (Figure 1). Obviously, the enrichment analysis for 5 selected genes by Kyoto Encyclopedia of Genes and Genomes (KEGG) and Reactome suggested involvement of these genes in pathways relevant to NDDs such as Huntington disease, neurotrophin signalling pathway and mitochondrial biogenesis (Figure 2A).

We attempted to find an appropriate miRNA according to two criteria listed below: a) targeting at least four genes in the PGC‐1α/FNDC5/BDNF pathway based on data retrieved from 3 prediction databases (TargetScan 7.1, miRmap and RNAhybrid) and b) dysregulation in NDDs based on literature survey. Collectively, literature review accompanied by computational analyses using various prediction software led us to select miR‐193b as an appropriate candidate for this study. Moreover, miR‐193b showed a differential expression between PD patients’ and healthy controls’ PBMCs in microarray data set (GSE16658) (Figure S1). To gain more insight into the importance of this miR in cellular processes, miRNA versus pathways heatmap was drawn. Interestingly, miR‐193b was indicated to be highly relevant to the pathways associated with the nervous system such as neurotrophin TRK receptor signalling pathway and neurodegeneration‐related pathways such as cell death, response to stress and intrinsic apoptotic signalling pathway (Figure 2B). The secondary structure of pre‐miR‐193b and potential binding sites of the miR within 3′UTR of five genes’ mRNAs implicated in PGC‐1α/FNDC5/BDNF pathway are depicted in the illustration (Figure S2).

### 3.3 Characterization of acute and chronic MPP+ in vitro PD models

To evaluate the effect of acute toxicity on SH‐SY5Y cells’ survival, the cells were exposed to several concentrations of MPP+ (0‐3000 µM) for 24 hours. According to MTS assay results, MPP+ significantly decreased cell viability in a concentration‐dependent way. 2000 µM MPP+ that caused a decrease by 42% in cell viability was chosen to induce acute oxidative stress in subsequent experiments (Figure S3B). SH‐SY5Y cells were also treated with the same doses of MPP+ 3 times per week for 14 days. Ratio of cell viability was 66% of that of untreated cells for SH‐SY5Y cells exposed to 1000 µM MPP+ which was finally selected as an optimal chronic dose (Figure S3B).

DCFH‐DA assay showed that there was a sevenfold rise in levels of reactive oxygen species (ROS) in acute PD model after 24‐hours MPP+ treatment. Likewise, chronic MPP+ exposure caused a remarkable increase in the number of DCF‐positive cells, reaching a peak of 77% after 2 weeks of chronic treatment (Figure S3C).

Also, fluorescein isothiocyanate (FITC)–labelled annexin V (Annexin V‐FITC) staining was used to measure apoptosis in the MPP+‐treated SH‐SY5Y cells. After 24 hours and 2 weeks of incubation with MPP+, staining for Annexin V clearly revealed that MPP+ significantly induced apoptosis in both acute and chronic in vitro PD models, rising from 15% and 21% to 36% and 52%, respectively (Figure S3D).

### 3.4 miR‐193b and PGC1a/FNDC5/BDNF pathway levels after acute and chronic MPP+ toxicity

**FIGURE 3:**
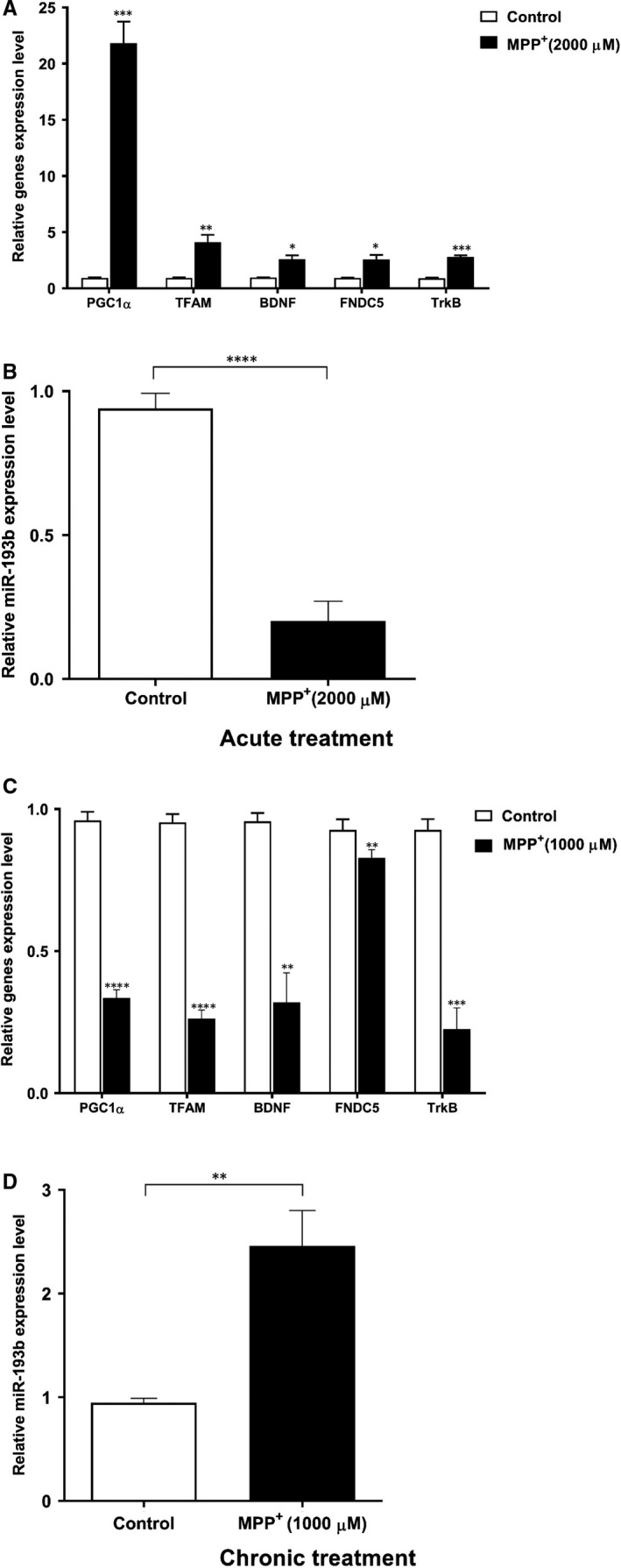
The effect of the acute and chronic MPP+ treatments on the expression of selected genes and miR‐193b. A, The predicted mRNAs levels were significantly up‐regulated in acute MPP+‐treated cells in comparison with the untreated cells. Expression of the target genes was normalized to GAPDH level as the reference gene. B, Acute MPP+ significantly down‐regulated miR‐193b expression level in SH‐SY5Y cells compared with the control cells. U6 was used to normalize the miR‐193b expression data. Data are represented as mean ± SD of three independent repeats of experiment. C, Administration of low‐dose repeated MPP+ significantly decreased mRNAs’ expression. D, miR‐193b was remarkably increased in the chronic MPP+ cellular model of PD. Data are represented as mean ± SD of three repeats of experiment (*P < .05, **P < .01, ***P < .001 and ****P < .0001 vs. control)

RT‐qPCR was used to assess the influence of acute MPP+ toxicity on PGC‐1α/FNDC5/BDNF pathway expression in SH‐SY5Y neuronal cells showing that mRNA levels were significantly affected and induced by acute toxicity for 24 hours in SH‐SY5Y cells compared to the untreated cells (Figure 3A). Data were normalized to GAPDH expression as a reference gene. By contrast, miR‐193b expression level was significantly declined in the acute MPP+‐treated cells compared with the control group (Figure 3B). Expression of miRNA was normalized to U6 snRNA regularly used as an internal control in miRNAs’ studies. Changes in the expression of miR‐193b and the predicted targets during chronic toxicity were also investigated by RT‐qPCR. Unlike acute model, administration of the low‐dose repeated MPP+ remarkably decreased mRNA level of PGC‐1α, TFAM, FNDC5, TrkB and BDNF compared with the untreated cells (Figure 3C), whereas miR‐193b expression was increased significantly at the end of the second week compared with control cells (Figure 3D).

### 3.5 miR‐193b overexpression effects on PGC‐1α/FNDC5/BDNF pathway level

**FIGURE 4:**
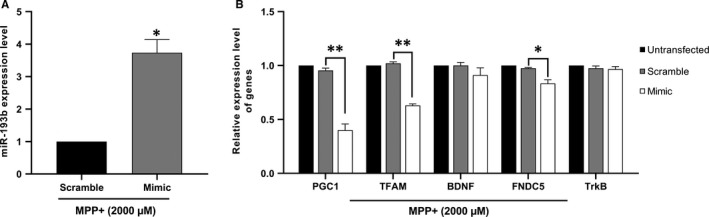
Mimic‐mediated overexpression of miR‐193b and suppressed expression of PGC‐1α/FNDC5/BDNF pathway. A, A significant rise was observed in the expression level of miR‐193b upon mimic transfection compared to the scramble group, confirming transfection efficiency. B, miR‐193b overexpression remarkably reduced PGC‐1α, TFAM and FNDC5 mRNA levels. Data are represented as mean ± SD of three repeats of experiment (*P < .05 and **P < .01 vs. scramble (negative control))

To check whether miR‐193b can be involved in MPP+‐induced alteration in PGC1a/FNDC5/BDNF pathway expression, miR‐193b has been overexpressed in SH‐SY5Y cells. Transfection efficiency was determined by qRT‐PCR, showing a significant more than threefold increase in miR‐193b level in the presence of miR‐193b mimic compared to the negative control (Figure 4A). To confirm the involvement of miR‐193b in MPP+‐induced toxicity, expression levels of PGC‐1α, BDNF, TFAM, FNDC5 and TrkB have been assessed following miR‐193b overexpression, resulting in the repression of PGC‐1α, TFAM and FNDC5 expression at mRNA levels (Figure 4B). Although a slight reduction has been observed in BDNF level, it was not statistically significant. Transfection of miR‐193b did not significantly affect TrkB levels as well.

### 3.6 Expression of miR‐193b and target genes in PD patients PBMCs

**FIGURE 5:**
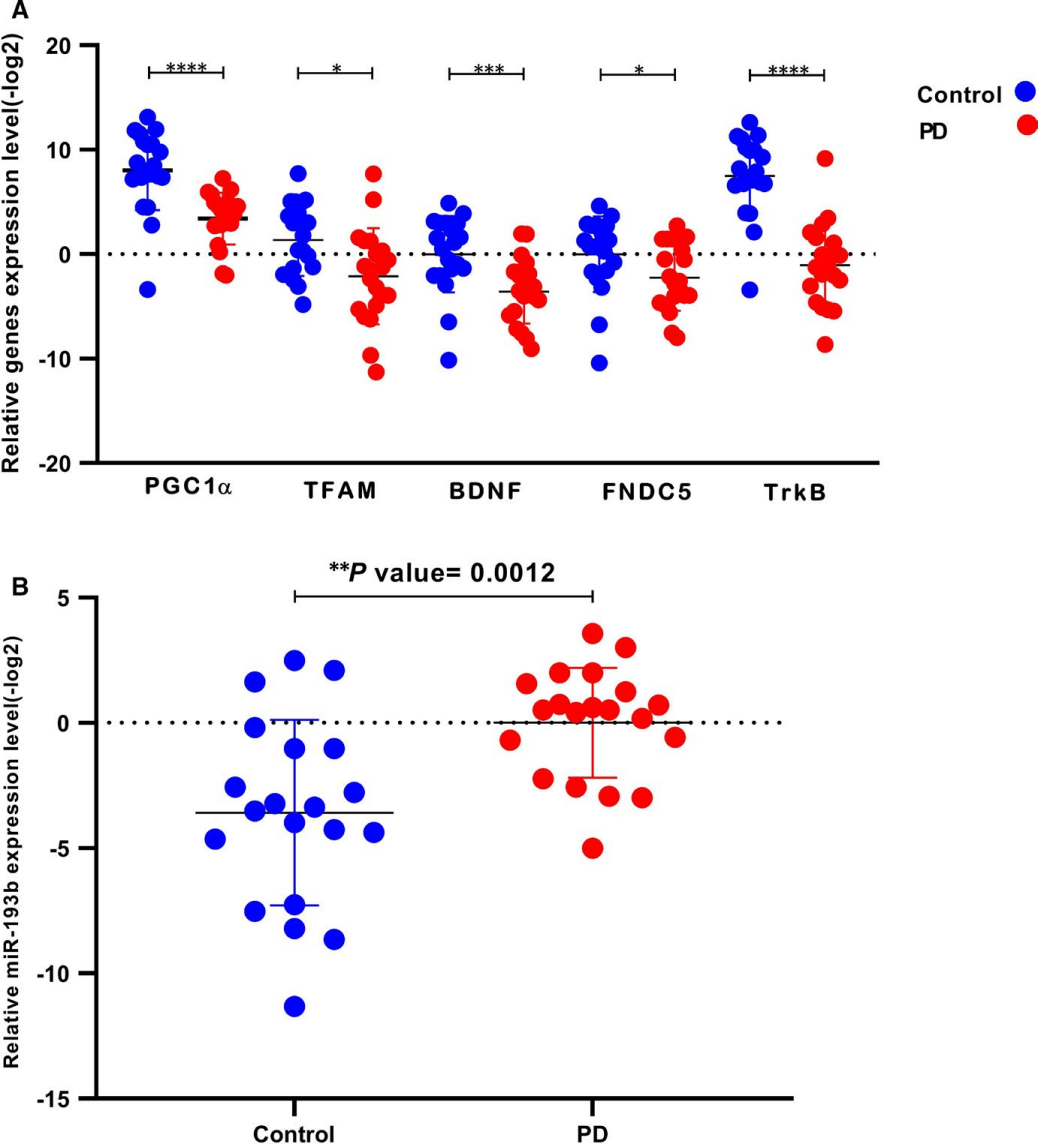
Selected genes and miR‐193b levels in PD PBMCs. A, A decrease occurred in PGC‐1α, TFAM, FNDC5, TrkB and BDNF levels in the PD patients in comparison with healthy controls. B, miR‐193b expression was increased in PBMCs from PD patients in comparison with healthy controls. Data are represented as mean ± SD (*P < .05, ***P < .001 and ****P < .0001 vs. control)

To investigate the expression of miR‐193b and predicted mRNAs levels in PBMCs of 20 PD patients and 20 healthy controls, RT‐qPCR analysis was performed. Similar to chronic PD model, there was a significant decrease in PGC‐1α, TFAM, FNDC5, TrkB and BDNF expression in PD PBMCs as shown in Figure 5A, while PBMCs of patients with PD showed an increased level of miR‐193b compared with controls (Figure 5B).

### 3.7 Diagnostic value of miR‐193b

**FIGURE 6:**
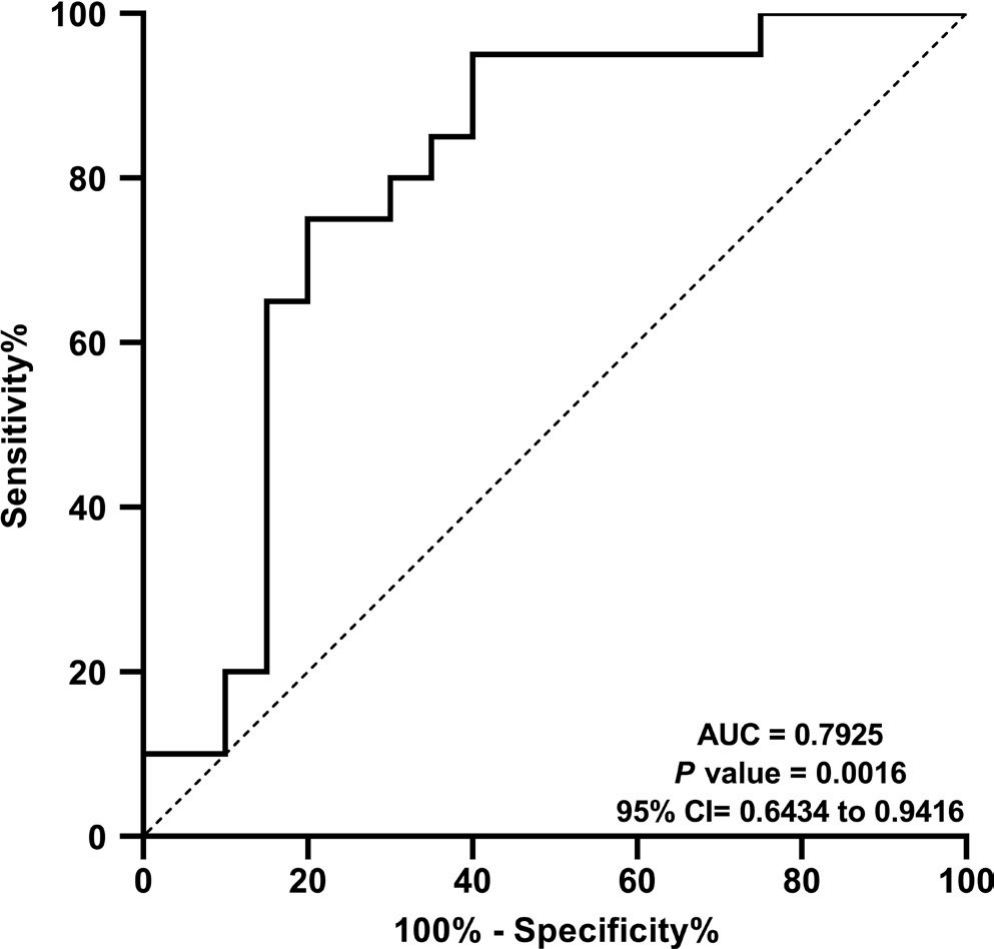
Diagnostic significance of miR‐193b. ROC curve analysis carried out to check sensitivity and specificity of miR‐193b revealed that the level of this miR can be a useful biomarker to distinguish PD patients and healthy individuals, with AUC value of 0.7925 [*P* = .0016, 95% CI: 0.6434‐0.9416]

The usefulness of miR‐193b in terms of differentiating PD patients from healthy controls, ROC curve and total area under the curve (AUC) were established. AUC value provides an estimate of the factor's potential to differentiate 2 groups by examining the sensitivity and specificity. The higher AUC value a marker has, the more effectively it can distinguish two conditions. The ROC curve analyses confirmed the significance of miR‐193b level as a potential biomarker for PD diagnosis, with AUC value of 0.7925 (*P* = .0016, 95% CI: 0.6434‐0.9416, Figure 6).

### 3.8 Correlation between expression levels of miR‐193b and PGC‐1α/FNDC5/BDNF pathway in PD PBMCs

**FIGURE 7:**
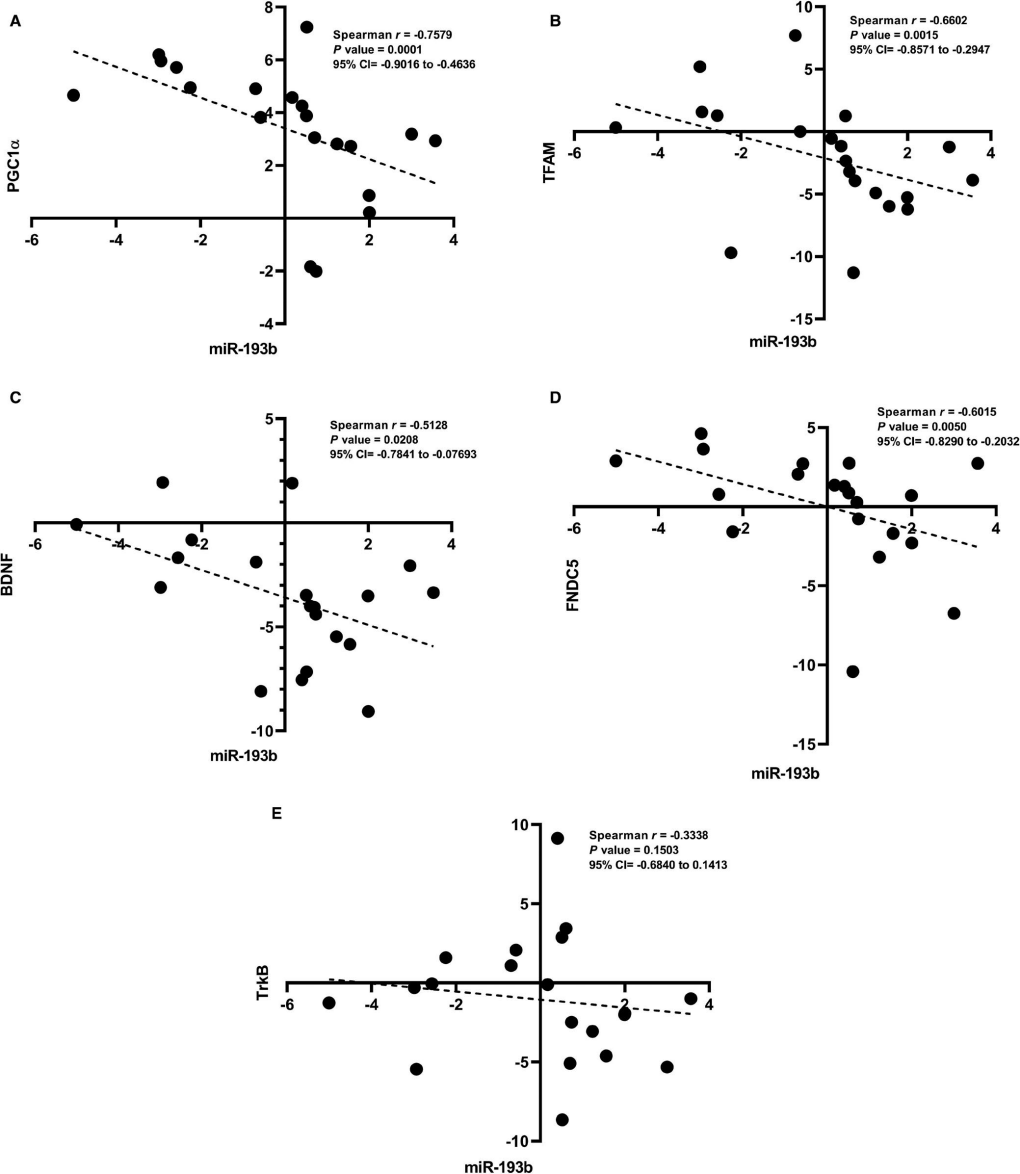
The inverse correlation between the increased miR‐193b and the decreased target genes expression in PD PBMCs. A, There was a statistically significant and strong inverse correlation between PGC‐1α and miR‐193b expressions. B‐D, Spearman's correlation test indicated moderate and significant opposite correlations between TFAM, BDNF, FNDC5 and miR‐193b expressions in PD PBMCs. E, No significant association was observed between TrkB and miR levels

Opposing expression patterns of miR‐193b with target genes in both cellular models and patients made us to hypothesize that there may be an association between expression levels of miR‐193b and PGC‐1α/FNDC5/BDNF pathway. In order to provide more insight into the relationship between miRNA‐mRNA pairs, statistical analysis was performed. While there was a statistically significant and strong inverse correlation between PGC‐1α and miR‐193b expressions (*r* = −.7579, *P* = .0001) (Figure 7A). Spearman's correlation test indicated moderate and significant opposite correlations between TFAM, BDNF, FNDC5 and miR‐193b expressions in PD PBMCs (*r* = −.6602, *P* = .0015/ *r* = −.5128, *P* = .0208/ *r* = −.6015, *P* = .0050, respectively) (Figure 7B‐D). However, no significant association was observed between TrkB and the miR levels (*r* = −.3338, *P* =.1503) (Figure 7E).

## 4 DISCUSSION

Although intensive research efforts have been made over the past decades to give insight into the pathomechanisms of PD, it still remains a major challenge in neurobiology.^6^, ^19^, ^36^ Inaccessibility of patients’ living target tissue and low availability or reliance on brain samples donated after death tend to impede significant advances in PD research, leading researchers to seek alternate windows into the biological basis of this disorder^20^, ^37^, that is why numerous useful in vitro systems have been previously established to provide PD researchers with appropriate replica for simultaneously studying the mechanism relevant to PD and screening of pro‐drugs.^2^, ^20^, ^38^ Growing evidence also represents that there is a correspondence between peripheral blood and brain tissue, suggesting that evaluating disease‐relevant genes’ expression in peripheral blood can act as an advantageous proxy measure for gene expression in the CNS and reflect the state of illness in the brain.^37^ PBMCs are deemed to be a peripheral laboratory providing a golden opportunity to study the mechanisms linked to NDDs and to find an indicator for their prognosis, diagnosis and treatment.^12^ ^,^ ^19^ Accordingly, here, cell models of PD were developed to clarify whether miR‐193b and PGC‐1α/FNDC5/BDNF pathway are affected by oxidative stress. Expression patterns of miR‐193b and potential target genes in PD patients’ PBMCs and miR‐193b's usefulness as a PD biomarker have also been examined.

MPP+ is a dopaminergic neurotoxin extensively utilized to reproduce PD in vitro to evaluate PD pathogenesis, physiology and efficacy of pharmacological agents.^10^, ^20^ Although acute MPP+ models have provided much valuable insight into many PD underlying mechanisms, they lack some pathological characteristics observed in PD progressive models and patients. Thus, some PD studies recently took advantage of chronic models with a continuous low‐level disturbance of mitochondrial and cellular function which provides a more accurate replication of the progressive nature of the disease.^39^ Thus, we made use of MPP+ to induce neurotoxicity in cultured neuronal cells, to construct both acute and chronic in vitro PD models. Previous studies have shown that oxidative stress and apoptotic pathways are two principal mechanisms of MPP+‐induced cell death.^2^ ^,^ ^19^, ^20^ Consistently, here, treatment of SH‐SY5Y neuronal cells with 2000 µM MPP+ for 24 hours and 1000 µM over 2 weeks remarkably increased the percentage of apoptotic cells and ROS generation, confirming that complex 1 has been blocked efficiently by MPP+ in both models of PD in our study.

Although studies have provided fragments of data about the potential link between PGC‐1α/FNDC5/BDNF pathway and NDDs, only a limited number of studies have investigated its expression in MPP+‐treated PD models and patients’ PBMCs. PGC‐1α is a highly conserved multifunctional transcriptional coactivator which has been suggested to exert protective effects against neurodegeneration.^4^, ^40^ Suppressed expression of PGC‐1α results in elevated α‐synuclein oligomerization and enhanced vulnerability of dopaminergic neurons to complex I inhibitors and α‐synuclein toxicity, while its stimulation appeared to improve dopaminergic neuronal viability in PD models and provides neuroprotection against α‐synuclein and neurotoxin‐induced damage^7^, ^9^, ^36^, Xue.^41^ Our results represented that PGC‐1α was strongly up‐regulated in acute PD model. Consistent with our data, one study showed that acute MPTP treatment regimen increased dramatically the expression levels of PGC‐1α in the striatum, cortex and cerebellum of mouse brain.^7^ PGC‐1α mRNA and protein expression were also increased in acute MPP+‐injured SH‐SY5Y compared with the control group, closely mirroring our result.^36^

TFAM is a target gene of PGC‐1α that regulates mitochondrial genome replication and transcription and its expression is associated with the mtDNA content.^11^, ^42^ There is also a mounting evidence for both direct and indirect implication of TFAM in PD pathogenesis via improving mitochondrial respiratory functions and providing protection against oxidative stress.^11^ TFAM overexpression inhibits ROS formation and reverses mitochondrial dysfunction in various in vivo and in vitro PD models, whereas TFAM inactivation culminates in parkinsonism phenotype accompanied by progressive motor dysfunction, inclusions’ accumulation and loss of dopaminergic neurons in mice SNpc.^11^, ^36^, ^38^ Here, acute MPP+ treatment remarkably induced TFAM expression level in SH‐SY5Y cells. Studies evaluating TFAM expression in disparate acute PD models have provided inconsistent data. TFAM has been previously identified to be induced by ROS in mammalian cells as a reactive factor to cellular oxidative stress, which is consistent with our results.^43^ Suliman et al also indicated that oxidative mtDNA damage induced by LPS results in elevated levels of mitochondrial TFAM.^44^ By contrast, according to Piao et al, both MPP+ and MPTP down‐regulated the expressions of TFAM at both mRNA and protein levels in SH‐SY5Y cells and mice brain, respectively.^10^

BDNF is the most studied neurotrophin that modulates a wide repertoire of neuronal functions including neuronal morphology, survival, growth and differentiation as well as synaptic plasticity through interaction with its high‐affinity receptor, TrkB.^12^, ^13^, ^14^, ^45^ BDNF/TrkB signalling axis is essential for the long‐term survival of nigrostriatal system whose impairment is frequently observed in PD, contributing to the onset and progression of the disease.^12^, ^14^ In vitro and in vivo studies found that BDNF treatment protects DA neurons from multiple neurotoxin insults in various PD models.^4^, ^12^, ^20^ Further, BDNF deficiency in CNS is known to have profound impacts such as synaptic dysfunction and impaired neuronal morphology, confirming the importance of this main trophic factor in various signalling pathways and circuits.^13^ In agreement with our data showing up‐regulation of BDNF in response to acute MPP+, BDNF levels rose considerably in C57BL6 mice striatum 24 hours after administration of high dose of MPP+, according to Cunha et al.^46^ By contrast, severe suppressed expression of BDNF along with its signalling axis regulator, TrkB, was clearly detected in acute MPTP‐induced PD mice as well.^14^

FNDC5 is a highly conserved transmembrane protein shown to be responsible for mediating the regulation of BDNF by PGC‐1α.^4^, ^16^ In neuronal culture, elevating the levels of FNDC5 was found to reverse the apoptotic effects of Ab1−42 oligomers in Alzheimer's disease (AD) model and counteracts the suppression of BDNF expression by them.^16^ However, knockdown of FNDC5 reduces BDNF expression in primary cortical neurons and impairs the development of neuronal precursors into mature neurons, representing an indispensable role of FNDC5 in neurons.^40^ In the current study, acute MPP+ toxicity induced expression of FNDC5 in neuronal cells. To the best of our knowledge, this is the first study to examine the level of FNDC5 in acute model of PD. Collectively, the observed rise in target genes’ levels can be a cellular adaptive response to MPP+ neurotoxicity to function as a compensatory protective strategy to attenuate destruction induced by neurotoxin.^7^ ^,^ ^19^

Notably, only a limited number of PD studies were carried out using chronic models, leading us to also assess changes in gene expression in neuronal cells treated chronically with MPP+. We identified a trend for decreased expression of all potential target genes in our chronic model of PD. The chronic low‐dose MPTP administration did not culminate in any significant change in PGC‐1α expression in one study.^7^ In contrast, striatal expression of PGC‐1α mRNAs was unexpectedly elevated in chronic mouse model of PD treated with MPTP for 5 weeks.^38^ Similar to the current study, Zhu et al showed that TFAM expression at the mRNA and protein level was significantly reduced in SH‐SY5Y cells treated with repeated low dose of MPP+ over 2 weeks.^47^ However, TFAM is reported to be unexpectedly up‐regulated in the striatum of chronic mouse model of PD injected with MPTP on a 5‐week schedule.^38^ In agreement with our findings, using APP/PS1 transgenic (Tg) mice as an AD experimental model, Xia et al reported markedly down‐regulated level of BDNF.^16^ Johnson et al observed significantly diminished levels of BDNF in the plasma of low‐dose rotenone model of PD as well. However, no change in full‐length TrkB was detected in their recent study using the same chronic experimental PD model.^48^, ^49^ Additionally, there was a dramatic drop in PGC‐1α and FNDC5 levels in response to Aβ toxicity in AD in vitro model.^16^ Moreover, 3 weeks after the injection of 6‐hydroxydopamine (6‐OHDA), a statistically significant decrease in mRNA level of PGC‐1α/FNDC5/BDNF signalling pathway in both striatum and hippocampus of rat was observed, exactly mirroring our findings.^50^

In spite of the most commonly applied acute models of PD whose induced impairments are temporary and spontaneously reversed several hours after toxin exposure, there are long‐lasting deficits in chronic models which more fully mimic progressive nature of the disease.^38^ ^,^ ^39^ Accordingly, here, suppressed expression of PGC‐1α /FNDC5/BDNF pathway showed the fact that damaged cells failed to protect themselves against toxicity after being exposed to repeated low‐dose MPP+, while SH‐SY5Y cells incubated with MPP+ acutely managed to immediately adapt to acute stress successfully by stimulating PGC‐1α /FNDC5/BDNF pathway components.

To verify the function of miR‐193b in MPP+‐induced toxicity, possible impacts of miR‐193b levels on 5 genes of PGC‐1α/FNDC5/BDNF pathway have been investigated. Expectedly, miR‐193b transfection had strong suppressive effects on PGC‐1α mRNA levels. FNDC5 and TFAM expressions have also been remarkably repressed as a result of miR‐193b overexpression. These findings not only confirm our bioinformatics predictions, but also suggest miR‐193b as a potential modulator of PGC‐1α/FNDC5/BDNF pathway in PD model.

Transcriptome of PD PBMCs as a beneficial representative of gene expression alteration in the CNS^12^, ^37^ was also assessed to more investigate the role of PGC‐1α/FNDC5/BDNF pathway in PD and its correlation with miR‐193b. Expressions of all five target genes were markedly lower in PD blood cells when compared with control PBMCs. Consistently, studies have revealed dramatic down‐regulation of PGC‐1α and its downstream‐regulated genes in human PD brain.^7^ PGC‐1α was also found to be diminished in AD PBMCs, while it was unchanged in mild cognitive impairment (MCI) patients’ blood cells. Interestingly, both AD and MCI PBMCs showed a significant decline of TFAM expression.^42^ Post‐mortem analysis of brains revealed down‐regulation of BDNF mRNA in the SNpc of PD‐affected patients as well as parietal cortex and hippocampus of AD patients.^13^, ^20^ On top of that, decreased peripheral level of BDNF/TrkB was observed in patients with PD which is deemed to be strongly associated with dopaminergic neurons neurodegeneration.^12^ Levels of truncated and full‐length TrkB are also decreased in various PD brain regions.^13^ To the best of our knowledge, this is the first study to assess peripheral expression of FNDC5 in various tissues of PD patients.

Increasing evidence indicates that miRNAs are implicated in PD pathogenesis by transcriptional regulation of PD‐linked pathways and genes. Some miRNAs have been recently proposed as biomarkers for various NDDs such PD and other conditions affecting the central nervous system^22^, ^23^, ^51^ ^,^ ^52^ Here, miR‐193b has been selected to be studied in 3 neurodegenerative conditions as a potential regulator of PGC‐1α/FNDC5/BDNF pathway, based on in silico studies as well as literature mining, indicating the aberrant expression of miR‐193b in various neurological disorders comprising amyotrophic lateral sclerosis (ALS)^53^, multiple sclerosis (MS)^54^ ^,^ Huntington's disease (HD)^21^ and AD.^51^ miR‐193b is a known mitomiR and redoximiR whose expression increases with age^55^, and has been introduced as a novel AD biomarker.^51^ In the present study, we observed a significant drop in miR‐193b expression after acute MPP+ treatment (2000 µM MPP+ for 24 hours), whereas chronic oxidative damage caused a dramatic rise in the miR’s level. In spite of confirmed association between miR‐193b and neurodegeneration, only a limited number of studies have evaluated its differential expression in models of NDDs. miR‐193b was previously introduced as an oxidative stress‐responsive miRNA, with its level being up‐regulated in primary hippocampal neurons exposed to hydrogen peroxide (H_2_O_2_).^52^ Moreover, expression of exosomal miR‐193b was diminished in the hippocampi, CSF like fluid and serum of mouse model of AD in comparison with the WT mice.^51^ Here, PBMCs of patients with PD showed increased expression of miR‐193b. Although there is not a report with regard to the significant dysregulation of miR‐193b in the PD patients, miR‐193a was shown to be increased in their plasma.^23^ In accordance with our findings, miR‐193b was one of the upderegulated miRNAs in the frontal cortex and the striatum of patients with HD.^21^ A rise in the exosomal miR‐193b level occurred in Parkinson's disease with dementia (PDD) patients’ serum compared with healthy individuals, although it was not statistically significant, as well.^22^ Additionally, microarray analysis identified miR‐193b as an up‐regulated miR in sporadic ALS patients’ leucocytes.^53^ By contrast, exosomal miR‐193b level was significantly reduced in serum of AD and MCI patients in comparison with that of control group.^22^ There were also lower levels of miR‐193b levels in serum and plasma of patients with MCI and dementia of Alzheimer type (DAT).^51^

In recent years, finding a promising biomarker for PD has been of utmost importance and blood miRNAs have always been one of the main considerations since they are not only easy handling and non‐invasive, but also more useful than cytokines and growth factors found in serum or plasma.^53^ Thereby, we evaluated diagnostic value of miR‐193b to differentiate PD‐affected patients from controls. Notably, the value of AUC for miR‐193b was satisfying (AUC = 07 925, 95% CI: 0.6434‐0.9416) and significant (*P* = .0016), suggesting that this miRNA might serve as a potential diagnostic biomarker in PD.

miR‐193b first showed opposite expression pattern with its possible target genes in all neurodegenerative conditions assessed in this study which can be considered as a first evidence validating our in silico results. Secondly, miR‐193b overexpression led to declined expression levels of PGC‐1α/FNDC5/BDNF pathway genes. Finally, possible correlations between miR‐193b and PGC‐1α/FNDC5/BDNF pathway components were further confirmed by Spearman's correlation coefficient data analysis. A statistically significant and strong inverse correlation was observed between PGC‐1α and miR‐193b in PD PBMCs (*r* = −.7579, *P* = .0001). There were also significant opposite associations between TFAM, BDNF and FNDC5 expression and miR‐193b level (*r* = −.6602, *P* = .0015/ *r* = −.5128, *P* = .0208/ *r* = −0.6015, *P* = .0050, respectively), providing further support for the hypothesis that changes in miR‐193b may affect PGC‐1α/FNDC5/BDNF pathway by regulating expression of PGC‐1α and other genes.

To sum up, our findings suggest that MPP+ administration at low doses can create a more appropriate model of PD in comparison with acute MPP+ toxicity. Deregulation of PGC‐1α/FNDC5/BDNF pathway was indicated in PD that may be a result of aberrant expression of miR‐193b, verifying the importance of PGC‐1α/FNDC5/BDNF pathway and miR‐193b as well as their associations in PD pathology. On top of that, this study provides evidence that miR‐193b expression in PBMCs may be a potential diagnostic biomarker for PD and give insights into the disease diagnosis. However, further studies must be conducted to confirm diagnostic value of miR‐193b and to fully comprehend mechanisms by which this miR operates on target genes in PD.

## CONFLICT OF INTERESTS

The authors declare that they have no competing interests.

## AUTHOR CONTRIBUTION

**Masoud Baghi:** Formal analysis (equal); Investigation (equal); Methodology (equal); Project administration (equal); Resources (equal); Software (equal); Writing‐original draft (equal); Writing‐review & editing (equal). **Elaheh Yadegari:** Formal analysis (equal); Investigation (equal); Methodology (equal); Resources (equal); Software (equal); Writing‐original draft (equal). **Mahsa Rostamian Delavar:** Formal analysis (equal); Investigation (equal); Methodology (equal); Project administration (equal); Validation (equal); Writing‐original draft (equal); Writing‐review & editing (equal). **Maryam Peymani:** Investigation (equal). **Mazdak Ganjalikhani‐Hakemi:** Formal analysis (equal); Resources (equal); Supervision (equal). **Mahri Salari:** Resources (equal); Supervision (equal); Validation (equal). **Mohammad Hossain Nasr‐Esfahani:** Project administration (equal); Supervision (equal). **Timothy Megraw:** Conceptualization (equal); Formal analysis (equal); Investigation (equal); Methodology (equal); Software (equal); Supervision (equal). **Kamran Ghaedi:** Funding acquisition (equal); Investigation (equal); Project administration (equal); Resources (equal); Writing‐review & editing (equal).

## Supporting information

Supplementary Material

## Data Availability

The data that support the findings of this study are available from the corresponding author upon reasonable request.
