# Supplementary material for: MiR‐193b deregulation is associated with Parkinson's disease
Source: J Cell Mol Med. 2021 May 20;25(13):6348–60. doi: 10.1111/jcmm.16612 (PMC8366452; doi:10.1111/jcmm.16612)
Supplement: Supplementary file 1 — Supplementary Material [file JCMM-25-6348-s001.pdf]

MiR-193b deregulation is associated with Parkinson's disease

Masoud Baghi[a,c*] (MSc.), Elaheh Yadegari[a*] (MSc.), Mahsa Rostamian Delavar[a*] (MSc.), Maryam Peymani[b,c] (PhD.), Mazdak Ganjalikhani-Hakemi[d] (PhD.), Mehri Salari[e] (MD.), Mohammad Hossein Nasr-Esfahani[c±] (PhD.), Kamran Ghaedi[a,c†±] (PhD.),

List of items:

Supplementary Figure 1. Differential expression of miR-193b in PD PBMCs.

Supplementary Figure 2. miR-193b as a promising candidate for targeting PGC-1α/FNDC5/BDNF pathway.

Supplementary Figure 3. Characterization of the acute and chronic *in vitro* PD models.

Supplementary Table 1. The primer-pair sequences of mRNAs for RT-qPCR

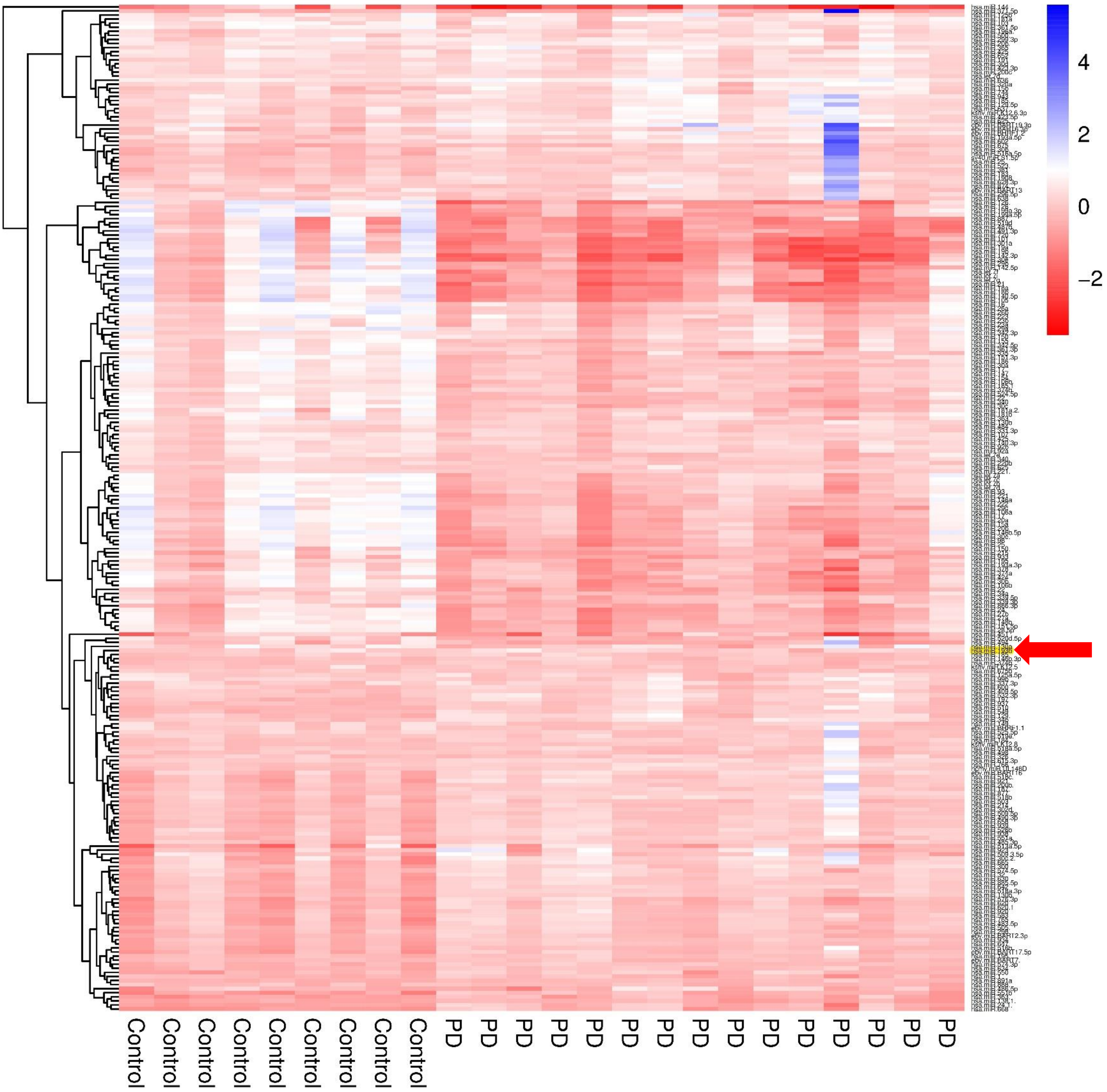
4
2
0
−2
Control
Control
Control
Control
Control
Control
Control
Control
Control
PD
PD
PD
PD
PD
PD
PD
PD
PD
PD
PD
PD
PD
PD
PD

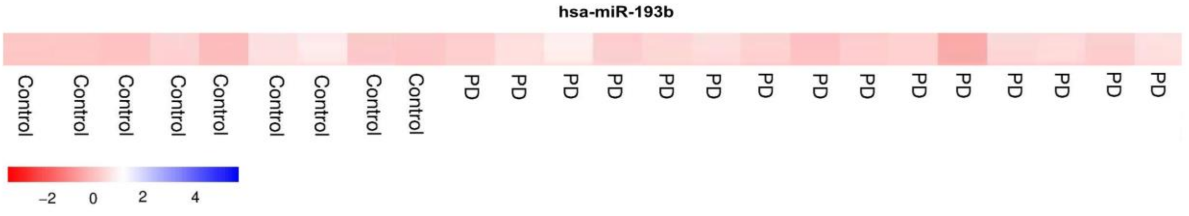
hsa-miR-193b
Control
Control
Control
Control
Control
Control
Control
Control
Control
PD
PD
PD
PD
PD
PD
PD
PD
PD
PD
PD
PD
PD
PD
PD
−2
0
2
4

Supplementary Figure 1. Differential expression of miR-193b in PD PBMCs. The heat-map panel indicates differentially expressed miRs, including has-miR-193b in Parkinson's diseases' PBMCs compared to healthy donors retrieved from microarray dataset (GSE16658).

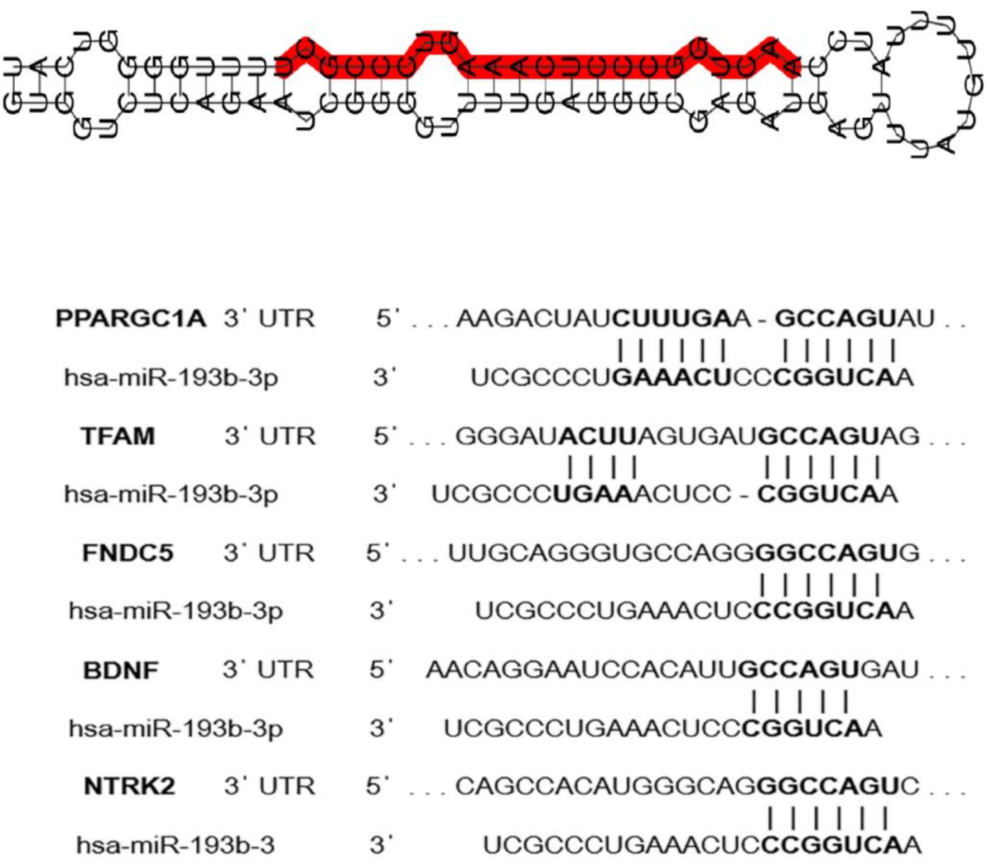

Supplementary Figure 2. miR-193b as a promising candidate for targeting PGC-1α/FNDC5/BDNF pathway. a) Secondary structure of pre-miR-193b retrieved from miRTarBase. Sequence of mature miR is shown in red. b) Complementarity sites for miR-193b in the 3' untranslated regions (3' UTR) of the predicted target genes, according to the TargetScan7.1 and miRmap databases. The bold letters indicate the predicted consequential pairing of target region (top) and miRNA (bottom).

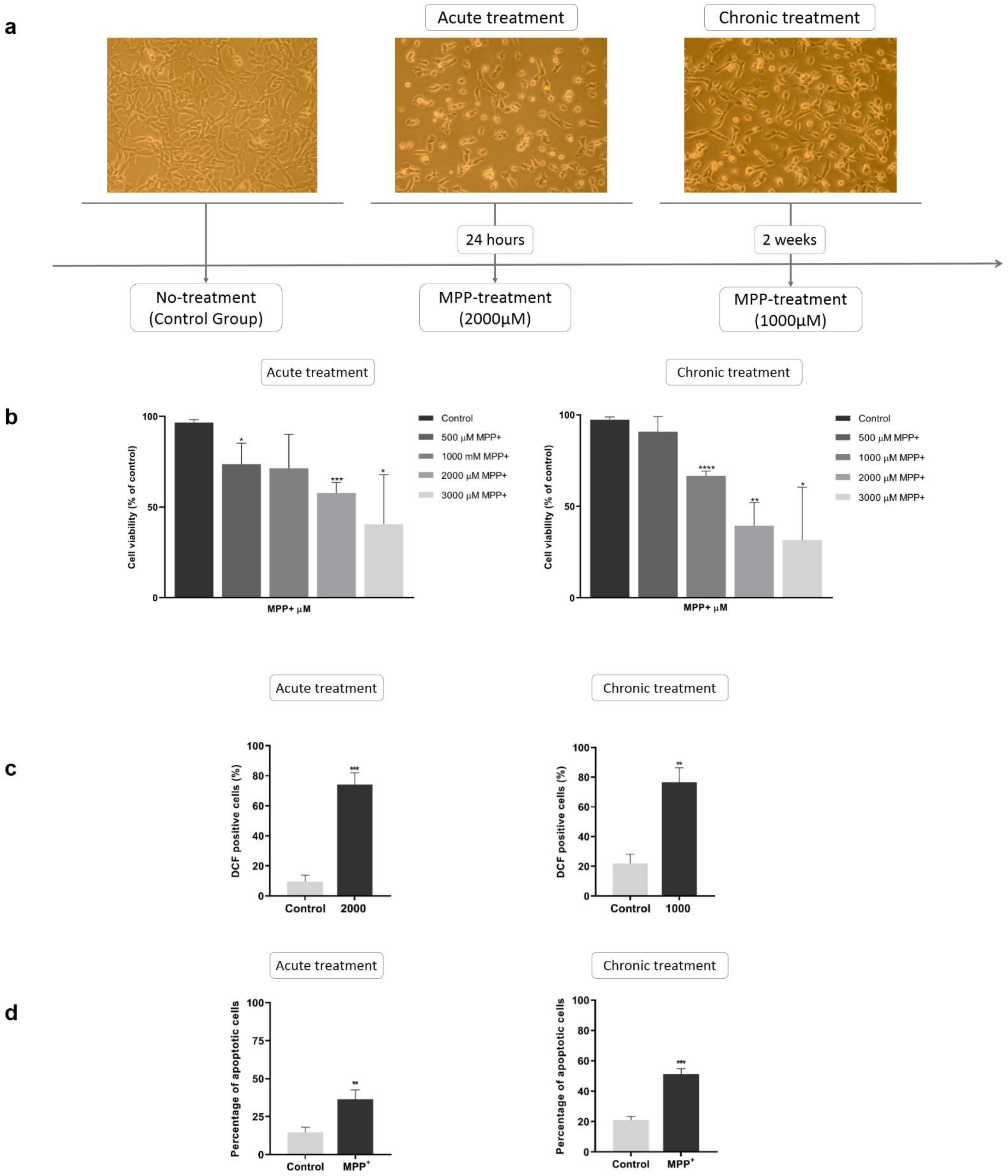

Supplementary Figure 3. Characterization of the acute and chronic *in vitro* PD models. a) Representative image of SH-SY5Y cells prior to MPP+ treatment exhibiting typical morphology (left), after acute (middle) and chronic (right) MPP+ treatment losing morphology. b) Cell viability assessment. SH-SY5Y cells were treated with 0-3000 µM concentrations of

MPP+ for 24 h (left) and 2 weeks (right). Cell viability was measured by MTS assay. 2000 µM and 1000 µM MPP+ were selected as optimal concentrations for creating acute and chronic models of PD which reduce cell viablility by 42% and 34%, respectively. c). ROS measurement by DCFH-DA staining. SHSY-5Y neuronal cells showed a nearly 7 and 4-fold increase in fluorescence intensity after acute (left) and chronic toxicity (right), respectively, showing increased generation of intracellular ROS following treatment with MPP+. d) Apoptotic cells detection using Annexin V-FITC staining. The statistical graph of Annexin V staining shwing that MPP+ induced apoptosis in both acute (left) and chronic (right) *in vitro* PD models, reaching 36% and 52% in acute and chronic PD models, respectively. (*$P < .05$, **$P < .01$ and ***$P < .001$ vs. control)

Supplementary Table 1. The primer-pair sequences of mRNAs for RT-qPCR

Gene	Sequence	Amplicon Size (bp)
PGC-1α	Forward: 5`- CCCCATGGATGAAGGGTACTT -3`<br>Reverse: 5`- GGGGAGGTCTCATCCATTGC -3`	138
TFAM	Forward: 5`-AATAGATAGGATGGGTTTGAG-3`<br>Reverse: 5`-AGATGACACAGGGACTTA-3`	131
FNDC5	Forward: 5`-AAGGGCAGATGTCAGCAATAC-3`<br>Reverse: 5`-TCAGCAGGGATGGAAGTCA-3`	86
BDNF	Forward: 5`-GCCTCCTCTTCTCTTTCTGCTG-3`<br>Reverse: 5`-CGCCGTTACCCACTCACTAATAC-3`	142
TrkB	Forward: 5`-GACTAAATCCAGCCCCGACAC-3`<br>Reverse: 5`-TCACAGACTTTCCTTCCTCCAC-3`	152
